# Where are the children in national hepatitis C policies? A global review of national strategic plans and guidelines

**DOI:** 10.1016/j.jhepr.2021.100227

**Published:** 2021-01-15

**Authors:** Farihah Malik, Heather Bailey, Polin Chan, Intira Jeannie Collins, Antons Mozalevskis, Claire Thorne, Philippa Easterbrook

**Affiliations:** 1 UCL Great Ormond Street Institute of Child Health, University College London, London, UK; 2 UCL Institute for Global Health, University College London, London, UK; 3 World Health Organization Regional Office for the Western Pacific, Manila, Philippines; 4 Medical Research Council Clinical Trials Unit, Institute of Clinical Trials and Methodology, University College London, London, UK; 5 WHO Regional Office for Europe, Copenhagen, Denmark; 6 Department of Global HIV, Hepatitis and STI Programmes, World Health Organization, Geneva, Switzerland

**Keywords:** Hepatitis C, Children, Adolescents, Pregnancy, Policy review, Policies, National strategic plans, Clinical practice guidelines, AASLD, American Association for the Study of Liver Diseases, APASL, Asian Pacific Association for the Study of the Liver, CPGs, clinical practice guidelines, DAAs, direct-acting antivirals, EASL, European Association for the Study of the Liver, ESPGHAN, European Society for Paediatric Gastroenterology Hepatology and Nutrition, GLE, glecaprevir, GHSS, Global Health Sector Strategy, GT, genotype, IDU, injecting drug use, IFN, interferon, LED, ledipasvir, LMICs, low- and middle-income countries, MoH, ministries of health, NASPGHAN, North American Society for Pediatric Gastroenterology Hepatology and Nutrition, NSPs, national strategic plans, PIB, pibrentasvir, RBV, ribavirin, SOF, sofosbuvir, VEL, velpatasvir, WHO, World Health Organization

## Abstract

**Background & Aims:**

It is estimated that 3.26 million children and adolescents worldwide have chronic HCV infection. To date, the global response has focused on the adult population, but direct-acting antiviral (DAA) regimens are now approved for children aged ≥3 years. This global review describes the current status of policies on HCV testing and treatment in children, adolescents, and pregnant women in WHO Member States.

**Methods:**

We identified national strategic plans and/or clinical practice guidelines (CPGs) for HCV infection from a World Health Organization (WHO) database of national policies from Member States as of August 2019. A standardised *proforma* was used to abstract data on polices or recommendations on testing and treatment in children, adolescents and pregnant women. Analysis was stratified according to the country–income status and results were validated through WHO regional focal points through August 2020.

**Results:**

National HCV policies were available for 122 of the 194 WHO Member States. Of these, the majority (n = 71/122, 58%) contained no policy recommendations for either testing or treatment in children or adolescents. Of the 51 countries with policies, 24 had specific policies for both testing and treatment, and were mainly from the European region; 18 countries for HCV testing only (12 from high- or upper-middle income); and 9 countries for treatment only (7 high- or upper-middle income). Twenty-one countries provided specific treatment recommendations: 13 recommended DAA-based regimens for adolescents ≥12 years and 6 still recommended interferon/ribavirin-based regimens.

**Conclusions:**

There are significant gaps in policies for HCV-infected children and adolescents. Updated guidance on testing and treatment with newly approved DAA regimens for younger age groups is needed, especially in most affected countries.

**Lay summary:**

To date, the predominant focus of the global response towards elimination of hepatitis C has been on the testing and treatment of adults. Much less attention has been paid to testing and treatment among children and adolescents, although in 2018 an estimated 3.26 million were infected with HCV. Our review shows that many countries have no national guidance on HCV testing and treatment in children and adolescents. It highlights the urgent need for advocacy and updated policies and guidelines specific for children and adolescents.

## Highlights

• Most national HCV policies lack specific recommendations for HCV testing and treatment in children and adolescents.
• Only 33 countries have specific recommendations for treatment of children and/or adolescents.
• Updated guidance on testing and treatment is needed for younger age groups.
• Efforts are particularly needed in countries with a high HCV burden in children.

## Graphical abstract

**Figure:**
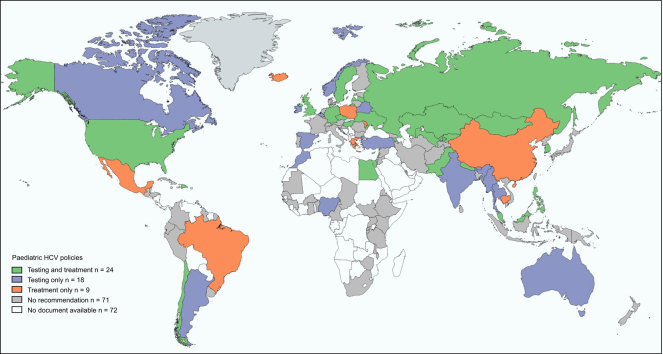

## Introduction

HCV infection is a major cause of chronic liver disease and associated morbidity and mortality worldwide.^1^^,^^2^ Globally, the World Health Organization (WHO) estimates that, in 2016, 71 million people were living with chronic HCV infection, with low- and middle-income countries (LMICs) disproportionately affected.^1^^,^^2^ In 2016, the WHO launched the Global Health Sector Strategy (GHSS) on viral hepatitis with a goal to eliminate viral hepatitis as a public health threat by 2030, defined as 65% and 90% reductions in hepatitis-related deaths and new infections respectively.^3^ The GHSS promotes Member States to develop national strategic plans (NSPs) to address viral hepatitis, as well as clinical practice guidelines (CPGs). Since this call to action, 5 WHO Regional Offices have developed region-specific plans for prevention and control of viral hepatitis,[4], [5], [6], [7], [8] and there has been a steady increase in both national plans as well as national guidelines for HCV testing and treatment.^9^

To date, the global scale-up of HCV screening and treatment has mainly been focused on adults, who bear the greatest burden of morbidity and mortality owing to chronic liver disease. Much less attention has been paid to testing and treatment strategies among children and adolescents. A recent systematic review estimated that 3.26 million (95% uncertainty interval 2.07–3.90) children and adolescents were living with chronic HCV infection in 2018 – 23 countries accounting for 80% of this global burden, and Pakistan, China, India, and Nigeria alone accounting for >50%.^10^

Transmission routes, disease progression and treatment indications in children differ from those in adults. Globally, vertical transmission is the principal HCV acquisition route among children^11^^,^^12^ but transmission also occurs through unsafe medical interventions, especially in LMICs.^13^ Adolescents may acquire infection through injecting drug use (IDU),^14^^,^^15^ and high-risk sexual practices especially among men who have sex with men.^16^^,^^17^ Although the occurrence of severe disease or cirrhosis in children is low at 2%, progression of liver disease occurs in childhood,[18], [19], [20] and can impact quality of life.^21^^,^^22^ Early diagnosis can help timely access to treatment and prevention of long-term morbidity.^23^

Before the availability of direct-acting antivirals (DAAs), there was limited treatment in selected children with interferon-based regimens which had low rates of viral clearance and significant side effects. DAA regimens have high rates of cure and minimal toxicity, and several regimens are now approved for use in adolescents and children, providing several opportunities to advance access to HCV care and treatment for children. First, the recent approval of several DAA regimens for use in adolescents (≥12 years), and now children aged >3 years^24^^,^^25^ and second, specific recommendations for testing and treatment in children and adolescents in guidance from WHO and 4 professional societies.^23^^,^[26], [27], [28], [29], [30], [31]

An assessment of national HCV policies may help identify where gaps exist regarding global guidance and professional society recommendations. Several surveys or reviews of viral hepatitis prevention and control programmes and policies have been undertaken at global^9^^,^^24^^,^^25^^,^^28^ or regional level, mostly in Asia and Europe.[32], [33], [34], [35], [36], [37], [38] Their scope ranged from gathering information about national hepatitis responses,^9^^,^^32^^,^^33^^,^^39^^,^^40^ to perspectives on treatment access and civil society engagement,[34], [35], [36], [37]^,^^41^ but none included questions specific to children or adolescents. There is also currently no systematic data collection or reporting of coverage of testing and treatment among adolescents and children.^2^

We undertook a global review of hepatitis C policies (both NSPs and CPGs) in WHO Member States, to identify specific policies and/or recommendations for testing and treatment in children and adolescents, and also of screening in pregnant women. We also reviewed international and professional society guidelines as the gold standard for comparison. Our aim was to identify areas of concordance and discordance in testing and treatment policies, and highlight critical gaps in advance of roll-out of DAAs for children.

## Materials and methods

### Search strategy, data sources, and data collection

#### WHO and major hepatology international and regional professional society guidance on HCV testing and treatment of chronic HCV in adolescents and children

We used guidelines from 5 professional societies (the American Association for the Study of Liver Diseases [AASLD], the Asian Pacific Association for the Study of the Liver [APASL], the European Association for the Study of the Liver [EASL], the European Society for Paediatric Gastroenterology, Hepatology and Nutrition [ESPGHAN], and the North American Society for Pediatric Gastroenterology, Hepatology, and Nutrition [NASPGHAN])[26], [27], [28]^,^^42^^,^^43^ and the WHO^30^^,^^44^ to identify those including relevant recommendations for adolescents and children.

#### NSPs and CPGs from WHO Member States

First, we identified NSPs and CPGs by searching for public documents available online (*e.g.* ministries of health (MoH) and national or regional hepatology/gastroenterology societies). Second, we performed a comprehensive search of PubMed to identify published guidelines in all languages. We used the search terms (‘Practice Guideline’ [Publication Type]) AND ‘Hepatitis C’ [MESH] from Jan 1, 2010 to Jun 30, 2019. Guidelines were found for 19 countries. Third, we searched the WHO repository of NSPs and CPGs on viral hepatitis to supplement the review.

Information on country hepatitis C policies was extracted from the identified documents. We searched each document for use of the following terms: ‘child’, ‘mother’, ‘pregnant’, and ‘adolescent’. If these terms were not explicitly mentioned, we searched for other related concepts, such as ‘age’, ‘young/youth’, and ‘birth’. We abstracted information on policies to address the following questions:

• Is there a policy on screening for HCV infection in pregnancy and, if so, is this routinely offered to all or targeted to specific high-risk groups?
• Is there a policy on testing in children and adolescents; if so, what are the criteria for testing and at what ages?
• What is the diagnostic algorithm for testing for HCV, and use of serological testing and use of HCV RNA?
• Is there a policy to treat children and adolescents; if so, at what age and with which regimens?

Documents available in languages other than English were translated into English using the Google Translate free online language translation service.

#### Questionnaire to 19 countries with high burden of paediatric HCV infection

To complement the above review, we conducted an online survey of HCV policies in the 19 countries with a high-burden of HCV, accounting for 80% of all paediatric HCV infections based on the 2016 estimates available at the time.^45^ These included Afghanistan, Algeria, Angola, Bangladesh, China, Democratic Republic of Congo (DRC), Egypt, Ethiopia, India, Indonesia, Iran, Iraq, Kazakhstan, Nigeria, Pakistan, Russian Federation, Syrian Arab Republic, Ukraine, and Uzbekistan. The semi-structured questionnaire focused on national HCV policies in pregnant women, children, and adolescents. Respondents were recruited through a purposive sampling process, and where possible, we identified MoH officials responsible for viral hepatitis. The survey was conducted using Research Electronic Data Capture, a web-based data collection tool.^46^

### Terminology

As the terms ‘policy’, ‘strategy’, ‘plan’, and ‘guideline’ are used in different ways across various settings, the following definitions were used for the purpose of this review: NSPs were defined as national strategies and action plans that provide overall strategic direction for a country’s response to hepatitis C, including those where viral hepatitis is an integrated part of broader health strategies. This definition was adapted from that used in the WHO manual for the development and assessment of national viral hepatitis plans^47^ and in a review of HCV policies in Europe.^38^ CPGs were defined as systematically developed statements designed to assist practitioners and patients in making decisions about appropriate healthcare (prevention, screening, diagnosis, testing, treatment and/or overall disease management) for individuals with HCV.^48^ The term policy was used to collectively refer to NSPs and CPGs. For this review, we defined an adult as a person aged ≥18 years, an adolescent as aged 12–17 years and a child as <12 years of age, unless stated otherwise, to conform with conventions in the literature.

### Data analysis

We tabulated country data according to WHO region and World Bank income country classification.^49^ Overall regional HCV burden in adults and in children was sourced from the Global Hepatitis Report^1^ and modelling estimates of paediatric HCV prevalence,^10^ respectively. We compared findings on availability of policies from countries with a high burden of paediatric HCV with other countries using Fisher’s exact test for categorical variables.

Data were collected January–June 2019 and validated through consultation with regional focal points in July to August 2020. We undertook internal validation of our results through cross-checking with findings from the recent policy review of Member States reported in 2019.^9^

### Data availability

The datasets used during the current study are available from the corresponding author on reasonable request.

## Results

### WHO and professional society guidelines

Recommendations from 2 WHO guidelines and 5 professional society guidelines for testing and treatment, relevant to children, adolescents, and pregnant women are summarised in Table 1, Table 2. As for adults, WHO recommends testing of high-risk children and adolescents (such as those exposed to medical interventions and treated in hospitals or those at risk of acquiring HCV sexually or through IDU), including those with a clinical suspicion of chronic viral hepatitis and children of infected mothers.^30^ Although professional society guidelines recommend testing children of infected mothers, most do not specify other groups of children and adolescents to be tested.^27^^,^^31^ The exception is the AASLD guidelines which recommend testing siblings of children with vertically-acquired HCV.^26^

**Table 1:** Comparison of international guidelines for screening and treatment of chronic HCV infection during pregnancy and in children and adolescents. Testing.

Guideline and year of publication	Screening in pregnancy	HCV testing of perinatally exposed children
American Association for the Study of Liver Diseases (AASLD) 2020^26^	All pregnant women should be tested for HCV infection, ideally at the initiation of prenatal care.	All children born to HCV-infected women should be tested for HCV infection. Testing is recommended using an antibody-based test at or after 18 months of age. Testing with an HCV-RNA assay can be considered in the first year of life, but the optimal timing of such a test is unknown. Repetitive testing by HCV RNA is not recommended. Children who are anti-HCV positive after 18 months of age should be tested with an HCV-RNA assay after age 3 years to confirm chronic hepatitis C infection. Siblings of children with vertically-acquired chronic HCV should be tested for HCV infection, if born from the same mother.
European Association for the Study of the Liver (EASL) 2020^27^	All pregnant women should be tested for HCV infection, ideally at early stages of pregnancy but testing can be carried out at any stage during pregnancy.	All children born to HCV-infected women should be tested for HCV infection from the age of 18 months.
North American Society for Pediatric Gastroenterology, Hepatology, and Nutrition (NASPGHAN) 2020^31^	No recommendation.	Antibody testing should be performed at 18 months of age. If requested by the family, the serum HCV RNA can be tested before 18 months of age; however, infants should be at least 2 months old.
World Health Organization (WHO) 2018^30^^,^^44^	Routine testing of all pregnant women for HCV infection is currently not recommended. Although, testing guidelines do recommend that information on risk factors for HCV infection should be communicated to pregnant women and that HCV testing should be considered alongside testing for HIV and HBV in high-endemic settings and for those with risk factors.	Infants whose mothers have been diagnosed with HCV should be followed up and routinely offered testing, and those diagnosed should be regularly monitored for signs of liver disease so that treatment can be offered when necessary. HCV infection in children under 18 months can be confirmed only by virological assays to detect HCV RNA, because transplacental maternal antibodies remain in the child’s bloodstream up until 18 months of age, making test results from serology assays ambiguous.

No recommendations: Asian Pacific Association for the Study of the Liver (APASL) 2016; European Association for the Study of the Liver (EASL) 2018; European Society for Paediatric Gastroenterology, Hepatology and Nutrition (ESPGHAN) 2018.

**Table 2:** Comparison of international guidelines for screening and treatment of chronic HCV infection during pregnancy and in children and adolescents. Treatment.

Guideline and year of publication	Treatment in pregnancy	Children and adolescents: who to treat?	Children and adolescents: drug regimens recommended
American Association for the Study of Liver Diseases (AASLD) 2020^26^	Treatment during pregnancy is not recommended owing to the lack of safety and efficacy data. For women of reproductive age with known HCV infection, DAAs recommended before considering pregnancy, whenever practical and feasible, to reduce the risk of HCV transmission to future offspring.	Treatment is recommended for all children ≥3 years old because they will benefit from antiviral therapy regardless of disease severity.	GT 1 and GT 4–6: weight-based sofosbuvir (SOF) and ledipasvir (LED) for children aged ≥3 years. GT 1–6: weight-based SOF and velpatasvir (VEL) for children aged ≥6 years or weighing ≥17 kg. GT 1–6: glecaprevir (GLE) (300 mg) and pibrentasvir (PIB) (120 mg) for adolescents aged ≥12 years or weighing ≥45 kg.
European Association for the Study of the Liver (EASL) 2018^27^	HCV treatment during pregnancy is not recommended in the absence of safety and efficacy data. However, treatment can be considered during pregnancy, or in the case of accidental conception during treatment, on a case-by-case basis. Treatment regimens for use in pregnancy not specified.	Treatment is recommended for all children ≥3 years.	GT 1–6: fixed-dose combination of SOF (400 mg) and VEL (100 mg) or fixed-dose combination of GLE (300 mg) and PIB (120 mg) for adolescents aged ≥12 years who are treatment-naive or treatment-experienced and without cirrhosis or with compensated (Child-Pugh A) cirrhosis. GT 1–6: weight-based, fixed-dose combination of SOF/VEL or GLE/PIB for children aged 3–11 years (some formulations pending approval).
European Society for Paediatric Gastroenterology, Hepatology and Nutrition (ESPGHAN) 2018^28^	No recommendation.	All treatment-naive and treatment-experienced children with chronic HCV infection should be considered for therapy. Treatment can generally be deferred in younger age groups for which combined peginterferon and ribavirin is the only treatment option currently available.	GT 1 and 4: children >12 years old or who weigh >35 kg should be given a combination of SOF (400 mg) and LED (90 mg) in a single tablet administered once a day for 12 weeks; recommended duration of therapy for treatment-experienced children with GT 1 infection and compensated cirrhosis is 24 weeks. GT 2: children >12 years old or who weigh >35 kg should be given SOF (400 mg) once a day plus weight-based RBV (15 mg/kg in 2 divided doses) for 12 weeks. GT 3: children >12 years old or who weigh >35 kg should be given SOF (400 mg) once a day plus weight-based RBV (15 mg/kg in two divided doses) for 24 weeks.
North American Society for Pediatric Gastroenterology, Hepatology, and Nutrition (NASPGHAN) 2020^31^	No recommendation.	Treatment is recommended for all children >3 years old.	Currently approved and anticipated DAA regimens (details not specified).
World Health Organization (WHO) 201830,44	Treatment is not recommended during pregnancy.	Treatment should be offered to all individuals diagnosed with HCV infection who are ≥12 years old, irrespective of disease stage.	GT 1 and 4–6: SOF (400 mg) and LED (90 mg) for 12 weeks. GT 2: SOF (400 mg) plus weight-based RBV for 12 weeks. GT 3: SOF (400 mg) plus weight-based RBV for 24 weeks.

No recommendation: Asian Pacific Association for the Study of the Liver (APASL) 2016.

DAAs, direct-acting antivirals; GT, genotype.

The 2017 WHO viral hepatitis testing guidelines,^30^ the AASLD,^26^ EASL,^27^ and the NASPGHAN guidelines,^31^ recommend serological testing in children only after 18 months of age, with detection of HCV RNA in those less than 18 months of age. Screening in pregnancy to identify children who could have been vertically exposed to HCV was only recommended by WHO and AASLD guidelines.

WHO (2018), AASLD, EASL, and ESPGHAN guidelines recommend use of sofosbuvir/ledipasvir or sofosbuvir/ribavirin for treating adolescents.[26], [27], [28]^,^^44^ All guidelines, except the recently updated EASL guidelines, currently recommend deferring treatment in younger children until interferon-free regimens are approved for younger age groups (Table 1, Table 2).

### National strategies and guidelines

NSPs or CPGs for hepatitis C were available for 122 of the 194 WHO Member States: 101 (83%) had NSPs, dated between 2010 and 2020, and 64 (52%) had clinical guidelines dated between 2009 and 2020 (Table 3). Of these, 43 countries (35%) had both NSPs and CPGs. The proportion of countries with national policies varied by region, with the highest in South-East Asia (n = 11/11, 100%) and the lowest in African region (n = 20/47, 43%). The proportion of countries with national policies also varied by World Bank income classification with 81% in high-income countries (46/57) compared with (17/31) 55% in low-income countries (Table S1).

**Table 3:** National policy recommendations for HCV screening during pregnancy, testing and treating children and/or adolescents, among countries with an NSP and/or guidelines.

	National policies in number (%) of countries						
		Screen all pregnant women	Screen some pregnant women	Screen children born to mothers with HCV	Test other children and/or adolescents for HCV	Treat children and/or adolescents for HCV∗	’Treat all’ but no policy specified for children
	Total n = 122	19 (16%)	13 (11%)	42 (34%)	5 (4%)	33 (27%)	20 (16%)
**By WHO region**							
	African n = 20	3 (15%)	1 (5%)	1 (5%)	0	0	0
	Eastern Mediterranean n = 10	3 (30%)	0	5 (50%)	0	3 (30%)	2 (20%)
	European n = 44	8 (18%)	8 (18%)	18 (41%)	2 (45%)	17 (39%)	10 (23%)
	Americas n = 22	3 (14%)	2 (9%)	5 (23%)	0	5 (23%)	5 (23%)
	South-East Asia n = 11	1 (9%)	1 (9%)	7 (64%)	1 (9%)	2 (18%)	2 (18%)
	Western Pacific n = 15	1 (7%)	1 (7%)	6 (40%)	2 (13%)	6 (40%)	1 (7%)
**By income classification**							
	High-income n = 46	4 (9%)	9 (20%)	17 (37%)	0	13 (28%)	12 (26%)
	Upper middle-income n = 32	6 (19%)	2 (6%)	11 (34%)	2 (6%)	10 (31%)	7 (22%)
	Lower middle-income n = 27	7 (26%)	1 (4%)	13 (48%)	2 (7%)	9 (33%)	1 (4%)
	Low-income n = 17	2 (12%)	1 (6%)	1 (6%)	1 (6%)	1 (6%)	0
	N/A	0	0	0	0	0	0

NSP, national strategic plan.

∗ Details of treatment regimens are shown in Table 5.

### Global overview of paediatric HCV policies

**Fig. 1:**
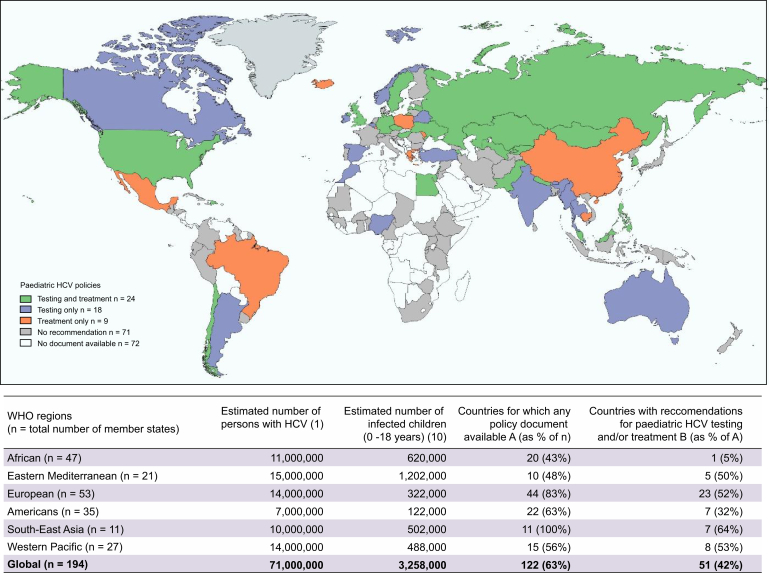
Countries with recommendations for paediatric HCV testing and treatment in national policy documents.

Fig. 1 shows the geographic distribution of countries that had recommendations to test and/or treat HCV-infected children and adolescents in national polices. Fifty-eight percent (n = 71) of the countries with available policies did not have any paediatric specific recommendations. Twenty-four countries had recommendations for both testing and treatment in children and/or adolescents (Armenia, Chile, Czech Republic, Dominican Republic, Egypt, Germany, Hungary, Kazakhstan, Republic of Korea, Kyrgyzstan, Latvia, Lebanon, Malaysia, Maldives, Mongolia, Nepal, Pakistan, Philippines, Russian Federation, Sweden, Ukraine, UK, USA, and Uzbekistan), and were mostly from the European region (n = 12) and were high- or upper-middle income (n = 16). Eighteen countries had policies only on testing, and again were more geographically diverse but mostly high- or upper-middle income (n = 12). Nine countries had policies on treatment only and the majority were high- or upper-middle income (n = 7).

### Policy landscape in 19 high paediatric burden countries

**Fig. 2:**
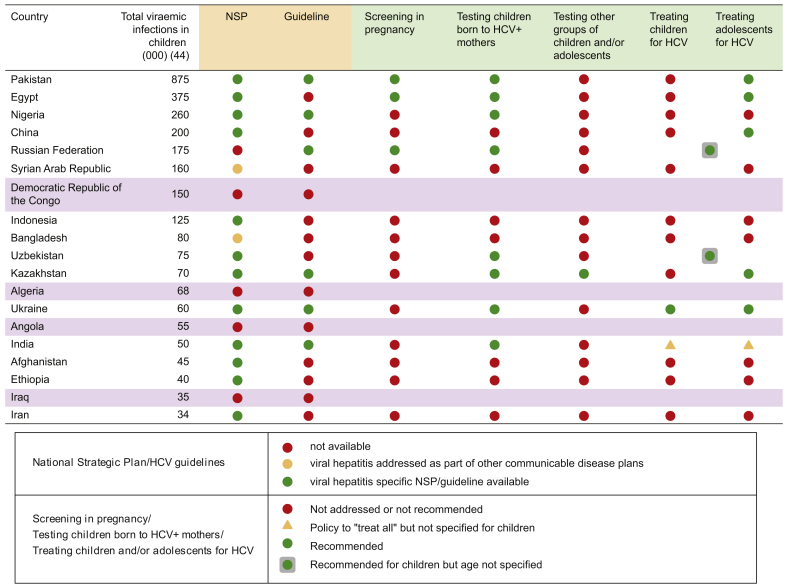
HCV policy landscape in countries that account for 80% of paediatric disease burden.

Fig. 2 shows the policy landscape in 19 countries that accounted for 80% of paediatric HCV burden in 2016 estimates.^45^ National policy documents were available for 15 of the 19 countries (9 NSPs, 1 CPG and 5 countries with both). Twenty percent (3/15) had recommendations on screening of pregnant women; 53% (8/15) on testing of children born to HCV-infected mothers; and 47% (7/15) on treating children and adolescents for HCV. There were no statistically significant differences in proportion with policies compared with non-high burden countries.

### HCV screening in pregnancy

Overall, of the 122 countries with policy documents available, most (n = 90, 74%) did not include any recommendation to screen for HCV during pregnancy. Of 32 countries with a recommendation, 19 countries recommended routine HCV screening for all pregnant women (Table 4), whereas in a further 13 countries, the national policy was targeted antenatal screening in high risk groups. This included HCV screening of pregnant women with clinical suspicion of infection and/or known risk factors such as IDU history (n = 11). Of the 32 countries recommending any form of screening in pregnancy, only 8 specified any service delivery details, such as how to test (first-line diagnostic test), when to test (*e.g.* first antenatal visit) and where to test, and these varied by country (Table 4).

**Table 4:** HCV screening during pregnancy, policy recommendations, and service delivery considerations.

Country	Screening policy	Policy details *How to test? When to test?*
Bahrain	All	HCV antibody test.
Cuba	All	HCV antibody test in the 3 trimesters of pregnancy.
Dominican Republic	All	RNA test during the third trimester of pregnancy.
Egypt	All	Establish viral hepatitis serologic surveillance among identified groups, including women receiving care at antenatal clinics.
Georgia	All	Details not specified
Ghana	All	Details not specified
Guinea	All	Details not specified
Hungary	All	Details not specified
Kyrgyzstan	All	Details not specified
Luxembourg	All	Details not specified
Republic of Moldova	All	Details not specified
Mongolia	All	Antibody test at the first antenatal visit (end of first trimester).
Myanmar	All	Details not specified
Pakistan	All	Details not specified
Romania	All	Details not specified
Russian Federation	All	Details not specified
Rwanda	All	At first antenatal visit.
Turkmenistan	All	Details not specified
USA	All	At the initiation of prenatal care.
Australia	At-risk	Details not specified
Belarus	At-risk	Details not specified
Belgium	At-risk	Details not specified
Cameroon	At-risk	Details not specified
Canada	At-risk	Details not specified
Greece	At-risk	Details not specified
Guatemala	At-risk	Details not specified
Ireland	At-risk	Details not specified
Nepal	At-risk	Details not specified
Norway	At-risk	Details not specified
Poland	In research settings	Details not specified
Spain	At-risk	Details not specified
UK	At-risk	HCV antibody testing in first or second trimester, with repeat test in third trimester for women with on-going risk factors with initial negative test.

### HCV testing in children and adolescents

#### Which children and adolescents to test

Only 42/122 (34%) countries had recommendations on testing of children born to HCV-infected mothers included in their policies and 5 (4%) outlined testing approaches for other groups of children or adolescents (those with clinical indications or history of hospitalisation). The diagnostic approaches recommended varied across countries (summarised in Table S2). Most of these countries did not have any pregnancy screening guidance (n = 23). Of the 32 countries recommending some form of screening in pregnancy, only 19 had a policy on testing of their infants.

#### When to test children of HCV positive mothers

In the 42 countries which recommended testing children of HCV-infected mothers, 30 included a specific recommendation on timing and choice of assay. Eight countries (Argentina, Australia, Belgium, Germany, Ireland, Myanmar, Russian Federation, and United Kingdom) recommended HCV RNA testing between 1 and 6 months of age, as the primary diagnostic test. Three countries (Bahrain, Kyrgyzstan, and Singapore) recommended use of antibody tests at 12 months, and 18 countries recommended antibody testing at 18 months or above. Only 6 countries (Argentina, Australia, Germany, Hungary, Ireland, and Kyrgyzstan) recommended different diagnostic algorithms for children based on the mother’s HCV viraemic status, with RNA testing recommended as the initial test for children of HCV-RNA-positive mothers and anti-HCV testing for infants of seropositive, HCV-RNA-negative women. Exceptionally, guidelines from Kyrgyzstan did not recommend testing in children born to seropositive mothers with a negative HCV RNA test; infants of HCV-RNA-positive mothers were recommended to undergo antibody testing at 12 months of age.

### HCV treatment in children and adolescents

Twenty countries had policies that included a broad recommendation to ‘treat all’ for HCV but without specific guidelines for treating children or adolescents. Another 33 countries specifically indicated that children and/or adolescents could be treated for HCV infection (Table 5).

**Table 5:** Treatment approaches in countries that recommend treating children and/or adolescents for HCV.

Country	Year	Paediatric treatment recommendations
Armenia		DAAs for 12–18-year-olds.
Brazil	2019	DAAs for 12–18-year-olds. IFN and RBV for 3–12-year-olds regardless of hepatic enzyme abnormalities.
Cambodia	2019	IFN and RBV for 2–18-year-olds. Treatment not recommended for children under the age of 2. HCV infected children to be monitored for liver disease progression.
Chile	2015	IFN and RBV for 2–18-year-olds. Treat GT 1 and GT 4 for 48 weeks; and GT 2 and GT 3 for 24 weeks.
China	2019	DAAs for 12–18-year-olds using: glecaprevir/pibrentasvir for all GTs with or without cirrhosis; sofosbuvir/ledipasvir for GT 1, 4, 5, and 6 and sofosbuvir and ribavirin for GT 2 and 3.
Czech Republic	2019	DAAs (sofosbuvir/ledipasvir) for 12–18-year-olds or those >35 kg. Defer treatment for those under 12 years of age until DAAs are available.
Dominican Republic	2018	DAAs for age groups in which DAA-based regimens are available. Treatment not recommended for children under the age of 3.
Egypt∗^,^†	n.a.	DAAs for 12–17-year-olds.
Germany	2018	DAAs for age groups in which DAA-based regimens are available. Treatment not recommended for children under the age of 3.
Greece	2017	DAAs for age groups in which DAA-based regimens are available. Treatment not recommended for children under the age of 3.
Hungary	2018	DAAs (sofosbuvir and ledipasvir) for 12–18-year-olds or those >35 kg IFN and RBV for 3–12-year-olds.
Iceland	2016	Treatment may be considered for 16–18-year-olds.
Kazakhstan	2019	DAAs for 12–18-year-olds. IFN and RBV for 3–12-year-olds in exceptional cases.
Korea, Republic of	2017	IFN and RBV for 3–18-year-olds
Kyrgyzstan	2011	Treatment for children indicated but regimen not specified.
Latvia	2017	IFN and RBV for 2–18-year-olds
Lebanon	n.a.	IFN and RBV
Luxembourg	2018	Treatment for children indicated but regimen not specified.
Malaysia	2019	DAAs for 12–18-year-olds using: sofosbuvir/ledipasvir for GT 1, 4, 5, and 6 and sofosbuvir and ribavirin for GT 2 and 3.
Maldives	2019	DAAs for 12–18-year-olds using: sofosbuvir/ledipasvir for GT 1, 4, 5, and 6 and sofosbuvir and ribavirin for GT 2 and 3.
Mexico	2016	IFN and RBV
Moldova, Republic of	2017	Treatment for children indicated but regimen not specified.
Mongolia	2019	DAAs for 12–18-year-olds using sofosbuvir/ledipasvir.
Nepal	2019	DAAs for 12–18-year-olds using sofosbuvir/ledipasvir.
Pakistan∗^,^†	n.a.	IFN and RBV (guidelines) Defer treatment for those under 12 years of age until DAAs are available.
Philippines	2020	DAAs for 12–18-year-olds using: sofosbuvir/velpastavir; glecaprevir/pibrentasvir; sofosbuvir/daclatasvir; sofosbuvir/daclatasvir.
Poland	2016	IFN and RBV for 3–18-year-olds
Russia∗	2016	Interferon-alpha AND ribavarin
Sweden	2018	DAAs for 12–18-year-olds using: sofosbuvir/ledipasvir 12 weeks for GT 1 or 4 and sofosbuvir/ribavirin 12–24 weeks for GT 2 or 3 *Option to use sofosbuvir/velpatasvir 12 weeks for GT 2 or 3*
Ukraine∗^,^†^,^‡	2016	IFN and RBV for 3–18-year-olds Treatment not recommended for children under the age of 3.
UK	2013	IFN and RBV
USA	2018	DAAs for age groups in which DAA-based regimens are available. Defer treatment for 3–12-year-olds until DAAs are available.
Uzbekistan∗	2011	IFN and RBV

DAAs, direct-acting antivirals; GT, genotype; IFN, interferon; n.a., not available; RBV, ribavirin.

∗ Indicates countries with a high paediatric HCV burden.^45^^,^^64^

† Survey response.

‡ Ukraine has since adopted WHO and EASL guidelines as the national guidelines.

#### Treatment in children

Nine country policies recommended treating children (minimum age of eligibility for treatment was 2–3 years); all recommended interferon/ribavirin-based regimens. An additional 4 countries recommended treatment with DAAs for age groups in which DAAs were available (without specifying the age groups).

#### Treatment in adolescents

Twenty national policies specifically recommended treating adolescents for HCV infection; 13 with DAAs, 6 with interferon/ribavirin-based regimens and 1 did not mention the regimen. Of note, the policies recommending DAAs were recent having been published between 2017 and 2020. Nine countries (China, Czech Republic, Hungary, Malaysia, Maldives, Mongolia, Nepal, Philippines, and Sweden) included details of DAA regimens for adolescents ≥12 years in the guidelines; sofosbuvir/ledipasvir was recommended by the majority. Some guidelines also included sofosbuvir/ribavirin, sofosbuvir/velpatasvir, and glecaprevir/pibrentasvir (Table 5).

## Discussion

Overall, this policy review showed that although many countries have made progress in developing national strategies for elimination of viral hepatitis, there are significant gaps in specific policies for testing and treatment in children and adolescents. Although 122 of 194 (63%) WHO Member States had NSPs and/or CPGs on HCV, of these, only 26% included recommendations on HCV screening in pregnancy and only 42% had any policies relating to testing and/or treatment for HCV in children and/or adolescents. Only 24 countries included recommendations for both testing and treatment of children in their national policies. As with other aspects of the global hepatitis response, little attention has been paid to designing policies that include children and adolescents, and country-level policies remain focused on adults.

### Who to test

Although the WHO recommends routine screening in pregnant women if the general population prevalence is ≥2%, or if pregnant women are from a high-risk group^30^ and the AASLD and EASL guidelines recommend testing all pregnant women,^26^^,^^27^ the majority of country policies and guidelines reviewed (74%) did not include any recommendation for routine or targeted HCV screening during pregnancy. Currently, the main purpose of testing for HCV in pregnancy is to identify those in need of treatment after delivery/breastfeeding for their own health benefit, as well as to prioritise testing of HCV-exposed infants. Routine testing of pregnant women is likely to expand if ongoing trials establish the safety of DAA treatment in pregnancy^50^^,^^51^ providing an opportunity for prompt treatment of mothers and prevention of HCV vertical transmission.

Although there was largely consensus in recommendations for testing and treatment of children and adolescents in WHO and professional society guidelines, there was heterogeneity in the recommendations in country policies. WHO recommends HCV testing for all children, adolescents, and adults at high-risk or with clinical suspicion of chronic viral hepatitis,^30^ and 3 professional society guidelines recommend testing of all children born to HCV-infected women.^26^^,^^27^^,^^31^ However, only 34% of the country policies reviewed recommended testing children born to HCV-infected mothers. Notably, other groups of children and adolescents potentially at high-risk (such as those exposed to medical interventions in settings with unsafe injecting practices) were only included in 5 country policies.

### Diagnostic algorithms for children born to HCV-infected women

WHO guidelines and professional society guidelines recommend testing children of infected mothers using HCV RNA in children <18 months of age serological tests after 18 months. Although 42 countries recommended testing children born to mothers with HCV, only 6 specified differential diagnostic approaches based on maternal serological and virological status. This lack of detail is consistent with findings from a recent survey of WHO Member States which reported that although most countries have testing policies, these are not necessarily comprehensive particularly regarding key populations.^9^ Methods to identify children with vertically-acquired HCV, and optimal timing of tests are needed given that the current screening and case identification practices are inadequate in pregnancy.^52^^,^^53^ Our findings also highlight a gap in other case-finding strategies for children, such as testing siblings of confirmed paediatric cases, who may also be at risk of vertical transmission or intra-familial transmission.

### Who to treat

The WHO and 4 professional society guidelines recommend deferring treatment for younger children until DAA regimens are approved for their age groups and treating children and adolescents for whom DAAs are licensed.[26], [27], [28]^,^^31^^,^^44^ These updated recommendations are not yet reflected in national policies as 9 country policies still recommended treating children with interferon/ribavirin-based regimens. Only 20 country policies specifically recommended treating adolescents for HCV infection.

### What drug regimens to use

Since 2018, several DAA regimens (sofosbuvir/ledipasvir, sofosbuvir/ribavirin, glecaprevir/pibrentasvir, and sofosbuvir/velpatasvir)^24^^,^^25^ have been approved for use in adolescents, and in September 2019 and March 2020, 2 DAA regimens were approved for use in children down to 3 years and 6 years of age.[54], [55], [56], [57], [58] WHO guidelines,^44^ as well as 3 professional society guidelines,[26], [27], [28] recommend sofosbuvir/ledipasvir, sofosbuvir/ribavirin, glecaprevir/pibrentasvir, and sofosbuvir/velpatasvir for treating adolescents. However, in this review only 13 countries recommended DAAs for treating adolescents. Sofosbuvir/ledipasvir was recommended in most guidelines, and only 3 specified use of other approved DAA regimens. Some national guidelines reviewed here pre-date both these WHO recommendations and licensure of DAAs for children; they therefore recommend older (interferon/ribavirin-based) regimens for children and there is a clear need for policies to be updated.

### Limitations of review

There were several limitations to this review. First was the challenge of accessing reliable and up-to-date information, as some national guidelines were under revision during the data collection process. When available, these unpublished/draft guidelines were reviewed. The policy situation may have since changed in some countries. National policy documents relating to overall paediatric and adolescent health which may have included reference to viral hepatitis were not reviewed. Our review only addressed current status of policies at the national level and not their level of adoption and implementation, which may lag.[59], [60], [61] It is therefore important to exercise caution in drawing conclusions about implementation and service delivery from the data included.

### What are the policy implications of our findings?

This review has demonstrated a ‘pregnancy and paediatrics policy gap’ in country-level policies, which needs to be addressed if global and regional elimination targets are to be achieved. Almost half of the countries with a high paediatric HCV burden lacked policy recommendations for testing or treatment of children and adolescents (7/15 for which policy documents were available). This highlights the need for further advocacy to ensure countries update their policies and guidelines to include HCV testing and treatment among children and adolescents and provide access to pan-genotypic DAA regimens. Data from studies in the USA indicate that even when maternal HCV infection is identified, most children at-risk of vertically-acquired HCV remain untested at 18 months of age.^52^^,^^53^^,^^62^ Current practices for paediatric case finding and linkage to medical care are inadequate and HCV-exposed children need to be identified systematically for appropriate counselling, testing, treatment, and follow-up. Absence of policies that outline case finding strategies for pregnant women and children contribute to the high proportion of children with HCV who remain undiagnosed.^52^

There is also the need for updating of WHO and professional society guidelines to include appropriate case-finding strategies in children and adolescents, and recommendations for use of DAA regimens in adolescents and younger age groups.^63^ This can be facilitated by leveraging existing antenatal and maternal and child health services to reach pregnant women and children, with a compilation of examples of good practices identified in case-finding and treatment of children. Recent progress in clinical studies of different DAA regimens in adolescents and children and regulatory approval together with development of paediatric formulations for those aged <6 years provide an opportunity to include children more comprehensively in the global hepatitis elimination response.

## Financial support

10.13039/100004423World Health Organization, Child Health Research Charitable Incorporated Organisation (CIO), and NIHR GOSH Biomedical Research Centre.

## Authors’ contributions

Conceived the project: FM, PE, CT. Extracted the data, wrote the first draft and made subsequent revisions to the manuscript: FM Designed the survey questionnaire: FM, HB, IJC, CT. Validated data: AM, PC. Reviewed results, provided guidance on methods, critically revised the paper, approved the final version to be published and agree to be accountable for all aspects of the work in ensuring that questions related to the accuracy or integrity of any part of the work are appropriately investigated and resolved: all authors.

## Data availability

The dataset used in this study is available from the corresponding author on reasonable request.

## Conflicts of interest

FM, AM, PC, HB, and PE declare no competing interests. CT has previously received grant funding from 10.13039/100010877ViiV Healthcare and BMS (through Penta Foundation). IJC reports grants from 10.13039/100006483Abbvie, 10.13039/100008021Bristol Myers Squibb, 10.13039/100016016Gilead, 10.13039/100008897Janssen Pharmaceuticals, and 10.13039/100010877ViiV Healthcare (through the PENTA Foundation).

Please refer to the accompanying ICMJE disclosure forms for further details.
