# Supplementary material for: Where are the children in national hepatitis C policies? A global review of national strategic plans and guidelines
Source: JHEP Rep. 2021 Jan 15;3(2):100227. doi: 10.1016/j.jhepr.2021.100227 (PMC7898178; doi:10.1016/j.jhepr.2021.100227)
Supplement: Multimedia component 1 [file mmc1.pdf]

Where are the children in national hepatitis C policies? A global review of national strategic plans and guidelines

Farihah Malik, Heather Bailey, Polin Chan, Intira Jeannie Collins, Antons Mozalevskis, Claire Thorne, Philippa Easterbrook

Table of contents

Table S1..2

Table S2..3

References...6

Table S1. Summary of national plans and clinical guideline documents reviewed, by WHO region and income classification

		All member states<br>A	Countries for which any policy document available<br>B (as % of A)	Both NSP and guidelines available<br>C (as % of B)	NSP only<br>D (as % of B)	Guideline only<br>E (as % of B)
**Total**		**194**	**122 (63%)**	**43 (35%)**	**58 (48%)**	**21 (17%)**
By WHO region:						
	African	47	20 (43%)	3 (15%)	16 (80%)	1 (5%)
	Eastern Mediterranean	21	10 (48%)	1 (10%)	7 (70%)	2 (20%)
	European	53	44 (83%)	21 (48%)	13 (30%)	10 (23%)
	Americas	35	22 (63%)	6 (27%)	12 (55%)	4 (18%)
	South-East Asia	11	11 (100%)	6 (55%)	4 (36%)	1 (9%)
	Western Pacific	27	15 (56%)	6 (40%)	6 (40%)	3 (20%)
By income classification:						
	High-income	57	46 (81%)	19 (41%)	17 (37%)	10 (22%)
	Upper middle-income	58	32 (55%)	11 (34%)	12 (38%)	9 (28%)
	Lower middle-income	46	27 (59%)	11 (41%)	14 (52%)	2 (7%)
	Low-income	31	17 (55%)	2 (12%)	15 (88%)	0
	#N/A	2	0	0	0	0

Table S2. Diagnostic pathways in countries that recommend testing children born to women with HCV

	Service delivery considerations for children born to women with HCV	
Country	**Testing recommendations** ***How to test? When to test? Where to test?***	**Follow up practices after initial test**
**Argentina**	HCV RNA test at age 3 to 6 months (If the mother has viraemia > 106 IU ml and/or coinfection with HIV, has a higher risk of transmission)	If RNA positive then repeat RNA after 3/4 months<br>If RNA negative then test antibody and RNA after 18 months
**Armenia**	Not specified	Not specified
**Australia**	HCV RNA test at age 2 months and again at 3-4 months later.<br>HCV antibody test at 18 months of age.	If both RNA tests are positive, referral to a Paediatric Gastroenterology or Infectious Diseases Unit for 6 monthly monitoring of liver function.
**Bahrain**	HCV antibody test after 12 months of age.<br>*Option to perform HCV RNA test at or after the infant's first well-child visit at age 1-2 months.*	Not specified
**Belarus**	Not specified	Not specified
**Belgium**	HCV RNA test at age 1 month.	Not specified
**Bhutan**	Not specified	Not specified
**Canada**	Not specified	Not specified
**Chile**	HCV antibody test after age 18 months.<br>*Option to perform HCV RNA test at age 3 months.*	If antibody positive, confirmation with HCV RNA test.
**Czech Republic**	HCV antibody test after age 18 months.	Not specified
**Dominican Republic**	HCV antibody test after age 18 months and two HCV RNA tests at an interval of 3-6 months.	Not specified
**Egypt***	Not specified	Not specified
**Germany**	HCV RNA test at age 6 months for children of **HCV RNA+ mothers**.<br>HCV antibody test at 15 months for children of **HCV antibody+, HCV RNA – mothers.**<br>*Tests for transaminase levels, RNA quantification and genotyping should be performed.*	Long-term follow up indicated but details not specified.
**Hungary**	HCV antibody test after age 18 months for children of HCV antibody+ mothers.<br>*Option to perform HCV RNA test at age 1-2 months.*	If antibody positive, refer to paediatric hepatologist and confirmation with HCV RNA test.

**India***	HCV antibody test after age 18 months. *Option to perform HCV RNA test after age 2 months.*	Not specified
**Ireland**	HCV RNA test at age 6 weeks and again at age 6 months for children of **HCV RNA+ mothers.** HCV antibody test after age 18 months for children of **HCV antibody+, HCV RNA- mothers.** No tests for children of **HCV antibody+, HCV RNA- women with assured eradication of infection and no ongoing risk for reinfection.**	If RNA negative on both occasions, test for HCV antibody after age 18 months.<br><br>If RNA or antibody positive, refer to Rainbow Clinic (specialist services).
**Kazakhstan**	Not specified	Not specified
**Korea, Republic of**	HCV antibody test after age 18 months. *Option to perform HCV RNA test after age 6 months.*	Not specified
**Kyrgyzstan**	HCV antibody test after age 12 months for children of HCV antibody+ and HCV RNA+ mothers. **No test for children of HCV antibody+, HCV RNA- mothers.** *Option to perform HCV RNA test after age 2 months.*	If antibody positive or born to mother with HCV/ HIV co-infection, confirmation with HCV RNA test.<br><br>If RNA positive, repeat RNA test.
**Latvia**	HCV antibody test after age 18 months. *Option to perform HCV RNA test after age 4 months.*	Not specified
**Lebanon**	HCV antibody test after age 18 months.	Not specified
**Malaysia**	HCV antibody test after age 18 months.	Not specified
**Maldives**	HCV antibody test after age 18 months. *Option to perform HCV RNA test between 0 - 12 months.*	Not specified
**Mongolia**	HCV antibody test after age 18 months. *Option to perform HCV RNA test between 0-12 months, preferably between 2-6 months.*	If antibody positive, confirmation with HCV RNA test at age 3 years.
**Morocco**	Not specified	Not specified
**Myanmar**	HCV RNA test at age 3 months.	If HCV RNA positive, repeat RNA test at 12 months to confirm.<br>If HCV RNA negative, monitor ALT levels every 3 months.
**Nepal**	Test after age 18 months.	Not specified
**Nigeria***	Not specified	Not specified
**Norway**	Not specified	Not specified
**Pakistan***	Not specified	Not specified
**Philippines**	HCV antibody test after age 18 months.	Not specified

**Russian Federation***	HCV RNA test at age 2 months and if positive, again at 2-6 weeks later.	If HCV RNA negative at age 2, 6, and 12 months AND antibody negative at age 12 months, remove from follow-up.<br>If HCV RNA negative at age 2, 6, and 12 months AND antibody positive at age 12 months, re-examine at 18 months.<br>If HCV RNA positive at 2 or 6 months and HCV RNA negative at 12 months, conduct HCV RNA and antibody tests at 18 and 24 months.
**Singapore**	HCV antibody test after age 12 months.	Not specified
**Spain**	HCV antibody test after age 18 months.<br>*Option to perform HCV RNA test.*	Not specified
**Sweden**	HCV antibody test after age 18 months.	If antibody positive, confirm with HCV RNA test.<br>If CHC confirmed, monitor liver function annually and HCV RNA quantification every 2-3 years.
**Thailand**	Not specified	Not specified
**Timor-Leste**	HCV antibody test after age 18 months.	If antibody positive, repeat antibody test or confirm with HCV RNA test.
**Turkey**	Not specified	Not specified
**Ukraine*#**	HCV antibody test after age 18 months.<br>*Option to perform HCV RNA test at age 2-18 months.*	If antibody positive, test HCV RNA.<br>If HCV RNA positive, repeat RNA test after 12 months to confirm.
**United Kingdom**	HCV RNA test at age 2-3 months.	Not specified
**United States of America**	HCV antibody test after age 18 months.<br>*Option to perform HCV RNA test during the first year of life, but optimal timing of such a test unknown.*	If antibody positive, confirm with HCV RNA test after age 3 years.
**Uzbekistan***	HCV antibody test after age 18 months.	Not specified

*indicates countries with a high paediatric HCV burden (1,2)

[#] Ukraine has since adopted WHO and EASL guidelines as the national guidelines.

References

1. El-Sayed MH, Razavi H. Global estimate of HCV infection in the pediatric and adolescent population. J Hepatol. 2015;62(Table 1):S831–2.

2. Razavi H, El-Sayed MH. Updated Global Estimate of HCV Infection in the Pediatric Population [Internet]. 2016. Available from: https://cdafound.org/content/Downloads/Pediatric Prevalence 161201.pdf
